# Essential Roles of RNA-binding Protein HuR in Activation of Hepatic Stellate Cells Induced by Transforming Growth Factor-β1

**DOI:** 10.1038/srep22141

**Published:** 2016-02-25

**Authors:** Jingjing Ge, Na Chang, Zhongxin Zhao, Lei Tian, Xianghui Duan, Lin Yang, Liying Li

**Affiliations:** 1 Department of Cell Biology, Municipal Laboratory for Liver Protection and Regulation of Regeneration, Capital Medical University, Beijing 100069, China

## Abstract

RNA-binding protein HuR mediates transforming growth factor (TGF)-β1-induced profibrogenic actions. Up-regulation of Sphingosine kinase 1 (SphK1) is involved in TGF-β1-induced activation of hepatic stellate cells (HSCs) in liver fibrogenesis. However, the molecular mechanism of TGF-β1 regulates SphK1 remains unclear. This study was designed to investigate the role of HuR in TGF-β1-induced SphK1 expression and identify a new molecular mechanism in liver fibrogenensis. *In vivo*, HuR expression was increased, translocated to cytoplasm, and bound to SphK1 mRNA in carbon tetrachloride- and bile duct ligation-induced mouse fibrotic liver. HuR mRNA expression had a positive correlation with mRNA expressions of SphK1 and fibrotic markers, α-smooth muscle actin (α-SMA) and Collagen α1(I), respectively. *In vitro*, up-regulation of SphK1 and activation of HSCs stimulated by TGF-β1 depended on HuR cytoplasmic accumulation. The effects of TGF-β1 were diminished when HuR was silenced or HuR cytoplasmic translocation was blocked. Meanwhile, overexpression of HuR mimicked the effects of TGF-β1. Furthermore, TGF-β1 prolonged half-life of SphK1 mRNA by promoting its binding to HuR. Pharmacological or siRNA-induced SphK1 inhibition abrogated HuR-mediated HSC activation. In conclusion, our data suggested that HuR bound to SphK1 mRNA and played a crucial role in TGF-β1-induced HSC activation.

Liver fibrosis is a common pathological wound-healing response to acute or chronic liver injuries of various causes^1^. It is characterized by the excessive accumulation of extracellular matrix (ECM), especially collagen α1(I) (Col α1(I)), which is predominantly produced by myofibroblasts (MFs). Hepatic stellate cells (HSCs) are the major source of MFs in injured liver.

Sphingosine kinase 1 (SphK1) catalyzes sphingosine phosphorylation to form sphingosine 1-phosphate (S1P), a lipid mediator with both intracellular and extracellular action modes. SphK1 and its product S1P regulate cell proliferation, differentiation, motility, survival and immune processes. Recently, numerous studies have demonstrated that SphK1 mRNA and protein expressions are significantly increased in the fibrogenesis of liver, cardiovascular system, lung, renal, and genital system^2^,^3^,^4^,^5^,^6^. In previous studies, we report that SphK1 expression and S1P production are increased in fibrotic liver of mouse and human^7^,^8^,^9^. Meanwhile, the liver injury is significantly decreased after SKI (a well-characterized SphK1 inhibitor) administration in carbon tetrachloride (CCl_4_)-treated mice, accompanied by a marked drop in mRNA levels of fibrosis markers, α-smooth muscle actin (α-SMA) and Col α1(I)^10^. These findings indicate that up-regulation of SphK1 plays a crucial role in liver fibrogenesis. Recent reports have illustrated that SphK1 expression is mainly up-regulated by transforming growth factor (TGF)-β1 in fibrogenesis^8^,^10^,^11^.

TGF-β1 is a major profibrogenic cytokine and plays a vital role in the activation of HSCs^1^. Particularly, TGF-β1 induces phenotypic transdifferentiation of HSCs and synthesis of ECM. Some studies have provided evidences that up-regulation of SphK1 is critical for the TGF-β1-induced fibrogenic effects^8^,^10^,^11^,^12^. Our previous studies show that TGF-β1 increases the expressions of Col α1(I) and α-SMA via up-regulating SphK1 in MFs^8^,^9^,^10^. When SphK1 expression is silenced by siRNA or its activity is inhibited by pharmacological inhibitor, TGF-β1-stimulated increase of Col α1(I) or α-SMA is significantly abolished. These observations prove that SphK1 plays a crucial role in TGF-β1-induced liver fibrogenesis. However, there are few reports about the molecular mechanism underlying TGF-β1-induced SphK1 expression. A recent report reveals that silence of Smad2, Smad3, or Smad4 blunts the TGF-β1-dependent increase of SphK1 protein level in C2C12 myoblasts, indicating that TGF-β1 up-regulates SphK1 expression through Smad signaling pathway^11^. Nevertheless, it is unclear whether other mechanisms are involved in TGF-β1-induced SphK1 expression.

Human antigen R (HuR), an RNA-binding protein, binds to U-rich or AU-rich elements (AREs), which are typically present in 3′ untranslated regions (3′UTRs) of target mRNAs^13^,^14^. HuR is predominantly located in nucleus and translocates to the cytoplasm while cells are stimulated by endogenous factors or external stimuli^15^. Some studies have revealed that HuR regulates target mRNA expression by prolonging mRNA half-life^16^,^17^,^18^. In addition, HuR affects the translation or subcellular distribution of target mRNAs^19^,^20^,^21^,^22^. HuR participates in a variety of physiological and pathological processes, such as cancer, inflammation, proliferation, angiogenesis, and apoptosis^23^. Recently, it has been reported that HuR mRNA level increases in activated HSCs isolated from livers of bile duct ligation (BDL)-treated mice and involves in TGF-β1-induced profibrogenic action^24^. Another study establishes that HuR binds to 3′UTR of TGF-β1 mRNA and regulates the fibrogenic response in cardiac fibroblasts triggered by TGF-β1 via a positive feedback circuit^25^. In addition, SphK1 mRNA and protein levels are increased after transfecting oncogene, v-Src, into mouse embryonic cells, and the increase is based on HuR-mediated mRNA stabilization^26^. All these observations provide us a clue that HuR might be an important player in the mechanism of TGF-β1-induced SphK1 up-regulation.

In the present study, we find that TGF-β1-induced HSC activation is HuR-dependent. In particular, we demonstrate that TGF-β1 promotes association of HuR with SphK1 mRNA and prolongs half-life of SphK1 mRNA by stimulating cytoplasmic accumulation of HuR. Our data represent the first experimental evidence about the relationship between HuR and SphK1 in HSC activation.

## Results

### HuR is up-regulated and translocates to cytoplasm during liver fibrosis

**Figure 1:**
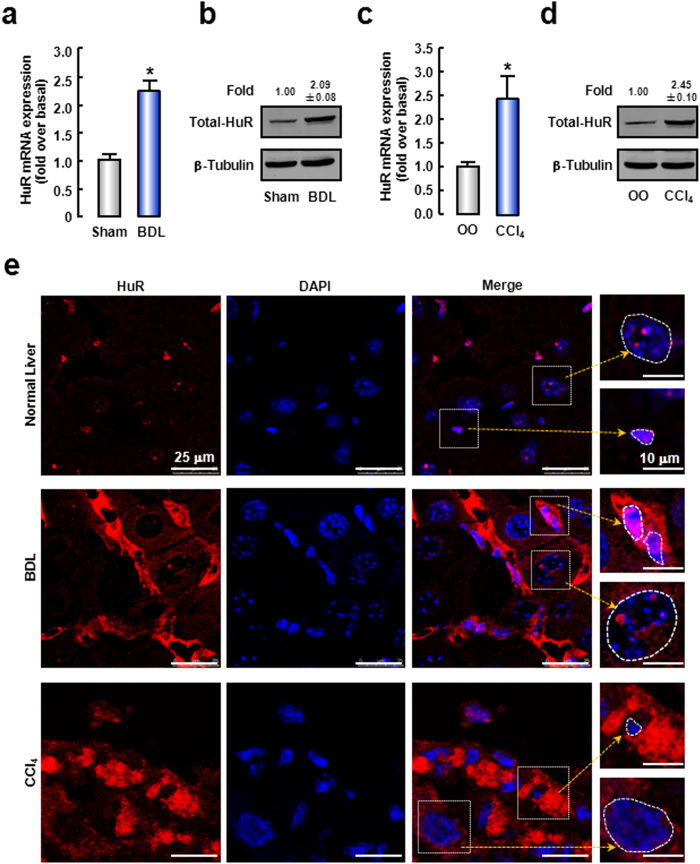
HuR is up-regulated and translocates to cytoplasm during liver fibrosis. (**a,b**) The expression of HuR mRNA (**a**) or protein (**b**) in fibrotic livers induced by BDL for 2 weeks. (**c,d**) HuR mRNA (**c**) or protein (**d**) expressions in fibrotic livers induced by CCl_4_ administration for 4 weeks. The cropped blots are used in the figure and full-length blots are presented in Supplementary Figure 5. Data are presented as the means ± SEM. *P < 0.05, versus the control livers. (n = 7, per group). (**e**) The representative images of immunofluorescence analysis by confocal microscopy to track HuR (red) in liver tissues of normal, BDL or CCl_4_ mice. DAPI was used to visualize nucleus (blue). Note that nucleus of hepatocytes (big circle) are several-fold larger than those of non-parenchymal cells (small circle).

First, we measured HuR expression in mouse fibrotic livers. The results showed that HuR mRNA and protein levels were significantly increased by ~2.20-fold and ~2.09-fold in the BDL-induced fibrotic livers, respectively (Fig. 1a,b). In CCl_4_-induced mice fibrotic livers, HuR mRNA and protein expressions were also increased by ~2.40-fold and ~2.45-fold, respectively (Fig. 1c,d). We also observed the expression of HuR in human fibrotic livers and found that the mRNA expressions of HuR were also increased in human fibrotic liver (Supplementary Figure 1a).

Next, we performed immunofluorescence analysis to examine the HuR localization in mouse fibrotic liver. As shown in Fig. 1e, in normal livers, HuR was predominantly located in the nuclei of hepatocytes and non-parenchymal cells. In BDL or CCl_4_-induced fibrotic livers, HuR protein expression was significantly increased, especially in cytoplasm of non-parenchymal cells. Moreover, the number of non-parenchymal cells was increased. The data indicate that cytoplasmic accumulation of HuR is increased in liver fibrogenesis.

### HuR correlates with fibrosis markers and binds to SphK1 mRNA in mouse fibrotic livers

Hepatic non-parenchymal cells include cholangiocytes, endothelial cells, Kupffer cells, HSCs and so on. HSCs, as one kind of non-parenchymal cells, are the key cells that activate as MFs and produce ECM. For this reason, we analyzed the expression correlation between HuR and fibrosis markers.

**Figure 2:**
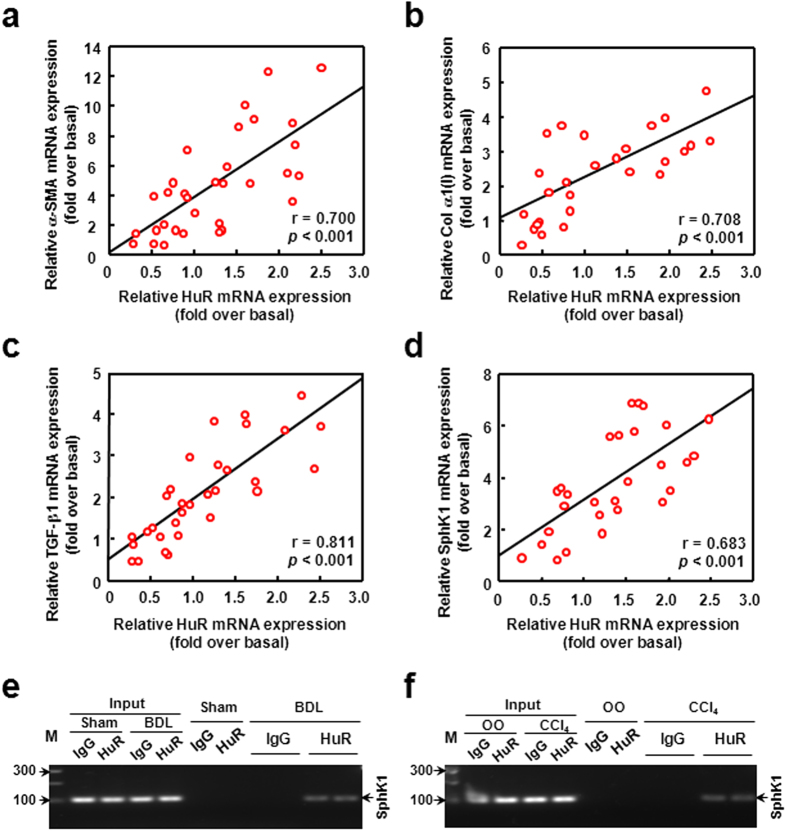
HuR closely correlates with fibrosis markers and binds to SphK1 mRNA in mouse fibrotic livers. The correlation between mRNA levels of HuR and α-SMA (**a**), Col α1(I) (**b**), TGF-β1 (**c**) or SphK1 (**d**) in mouse fibrotic livers. (**e**) Liver non-parenchymal cells of Sham or BDL mice were isolated and RIP analysis was performed. SphK1 mRNA was measured by qRT-PCR, and the PCR products were size-fractionated in a 2% agarose gel. The gels are run under the same experimental conditions. (**f**) Liver non-parenchymal cells of mice treated with OO or CCl_4_ were isolated and RIP analysis was performed as described.

In our previous study, we have reported that the expressions of α-SMA and Col α1(I) (markers of the activation of HSCs) are dramatically up-regulated in mouse liver fibrogenesis^27^. In human fibrotic livers, we also detected the up-regulations of α-SMA and Col α1(I) mRNA expressions (Supplementary Figure 1c,d). Here we undertook correlation analysis between mRNA levels of HuR and the two markers in mouse liver tissues. As shown in Fig. 2a,b, the mRNA levels of HuR had positive correlations with α-SMA (r = 0.700, *P* < 0.001) and Col α1(I) (r = 0.708, *P* < 0.001). In addition, it has been verified by our earlier report that TGF-β1 (a key profibrotic cytokine) expression is marked increased in fibrotic liver^10^. We undertook correlation analysis between HuR and TGF-β1 mRNA levels in liver tissues. HuR mRNA level had a positive correlation with TGF-β1 mRNA (r = 0.811, *P* < 0.001) (Fig. 2c). Furthermore, HuR mRNA expression also had a positive correlation with SphK1 mRNA, (r = 0.683, *P* < 0.001) (Fig. 2d). Notably, we also detected the up-regulation of SphK1 mRNA expression in human fibrotic livers (Supplementary Figure 1b).

To determine the relationship between HuR and SphK1 *in vivo*, we isolated the hepatic non-parenchymal cells and performed RNA immunoprecipitation (RIP) analysis to detect the binding between HuR and SphK1 mRNA. We found that the association between HuR and SphK1 mRNA was significantly enhanced in BDL-induced fibrotic liver (Fig. 2e). Moreover, this phenomenon was also observed in the livers of CCl_4_-treated mice (Fig. 2f). As HSCs are the key cells that produce ECM, we focused on HSCs to study the role of HuR in liver fibrogenesis. First, we detected whether HSCs were included in the non-parenchymal cells we prepared. The results of RT-PCR showed that α-SMA (the marker of MFs) mRNA was detectable and up-regulated in the non-parenchymal cells of fibrotic livers, indicating the presence of MFs (activated HSCs) in the non-parenchymal cells we prepared (Supplementary Figure 2a). Then we isolated primary mouse HSCs from normal or fibrotic livers. In primary HSCs, HuR expressed and localized in the nuclei of HSCs (Supplementary Figure 2c). Furthermore, the binding between HuR and SphK1 mRNA was also detected in the primary HSCs isolated from fibrotic livers (Supplementary Figure 2b). These data demonstrate that HuR binds to SphK1 mRNA in liver fibrogenesis.

Collectively, these results suggest that HuR interacts with SphK1 and might play an essential role in liver fibrogenesis.

### TGF-β1 induces activation of HSCs by increasing cytoplasmic accumulation of HuR

To further investigate the role of HuR in liver fibrogenesis, we employed LX-2, an immortalized low-passaged human HSCs line derived from normal HSCs and which exhibits the typical features of HSCs in primary culture.

We first examined the response of LX-2 to TGF-β1 treatment. LX-2 was treated with 10 ng/mL TGF-β1 and collected at 0, 4, 12, and 24 hours. qPCR analysis showed that the mRNAs of SphK1, α-SMA and Col α1(I) were up-regulated from 4 hours with a maximal increase at 24 hours (Supplementary Figure 3a). Their mRNA levels were also detected in LX-2 treated with 0, 1, 2, 5, or 10 ng/mL TGF-β1 for 24 hours. The results showed that the mRNA levels of SphK1, α-SMA and Col α1(I) were up-regulated in a dose-dependent manner (Supplementary Figure 3b). For these reasons, we treated LX-2 with 10 ng/mL TGF-β1 for 24 hours in our further experiments.

**Figure 3:**
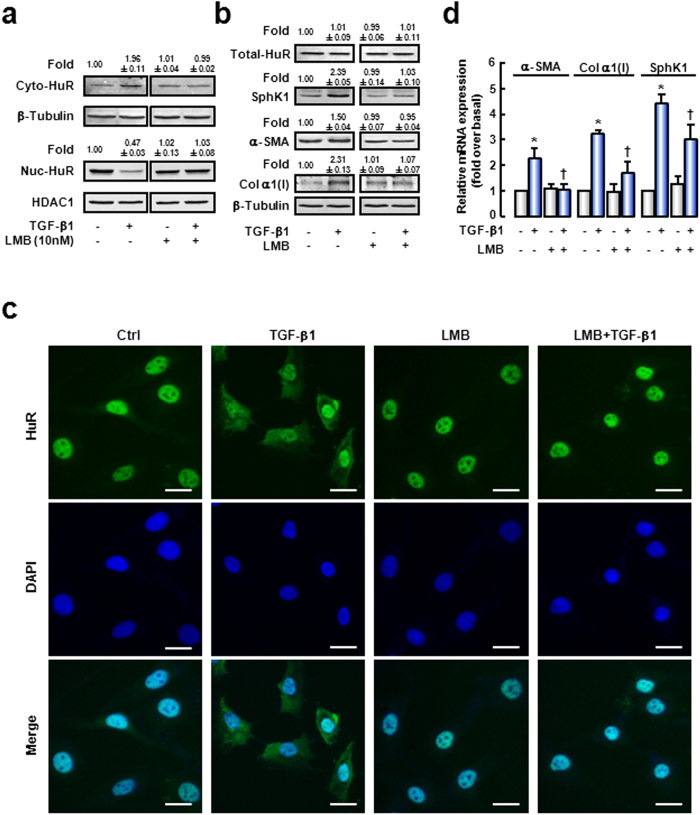
TGF-β1 induces activation of HSCs by increasing cytoplasmic accumulation of HuR. LX-2 were pre-treated with LMB (10 nmol/L) for 2 hours and followed by TGF-β1 (10 ng/mL) treatment for 24 hours. HuR levels in cytoplasmic or nuclear lysates (**a**), and total HuR, SphK1, α-SMA and Col α1(I) levels (**b**) were evaluated by Western blot analysis. The cropped blots are used in the figure and full-length blots are presented in Supplementary Figure 6. The levels of β-Tubulin (a cytoplasmic protein) and HDAC1 (a nuclear protein) in the same samples were assessed to ascertain the quality of the fractionation procedure and to detect loading differences. The intensity of each band was quantified and normalized to β-Tubulin. The values were the mean intensity normalized of each band (fold over basal). (**c**) HuR cytoplasmic accumulation was evaluated by immunofluorescence. DAPI was used to visualize nuclei (blue). (**d**) SphK1, α-SMA or Col α1(I) mRNA expression was examined by qRT-PCR analysis. Data are presented as the means ± SEM derived from at least three independent experiments. *P < 0.05, versus untreated control cells. ^†^*P* < 0.05, versus cells treated with TGF-β1 alone. Scale bars = 25 μm.

HuR cytoplasmic accumulation is considered as a prerequisite for its function. So we detected HuR localization in the presence of TGF-β1. As shown in Fig. 3a (left panel), cytoplasmic HuR protein was increased in TGF-β1-treatment cells, while nuclear HuR was decreased. If the ratio of cytoplasmic to nuclear HuR (Cyto/Nuc) in untreated cells was set as 1.00, the Cyto/Nuc was 4.17 in TGF-β1-treated cells (Table 1). Meanwhile, total HuR expression was not changed by TGF-β1 treatment (Fig. 3b). Moreover, LMB, a specific nuclear export inhibitor, was administrated in LX-2 culture medium before TGF-β1 treatment. The results of Western blot (Fig. 3a, right panel; Table 1) revealed that TGF-β1-induced HuR cytoplasmic accumulation was reduced after LMB pre-treatment (Cyto/Nuc = 0.96), while LMB had no effect on the total HuR protein expression (Fig. 3b). These data demonstrate that LMB blocks TGF-β1-induced cytoplasmic accumulation of HuR. Immunofluorescence analysis showed similar results (Fig. 3c).

Next, we examined whether cytoplasmic translocation of HuR involved in TGF-β1-induced HSCs activation. In TGF-β1-treated cells, protein expressions of α-SMA (~1.50-fold) and Col α1(I) (~2.31-fold) were increased (Fig. 3b, left panel). The increases were strongly attenuated when HuR cytoplasmic translocation was blocked by LMB (Fig. 3b, right panel). The mRNA levels of α-SMA and Col α1(I) were also examined (Fig. 3d). Consistent with protein expression, TGF-β1 triggered up-regulations of α-SMA (~2.27-fold) and Col α1(I) (~3.24-fold) mRNA expressions. Exposure of cells to LMB significantly blocked this effect.

Altogether, our results demonstrate that TGF-β1 induces HuR cytoplasmic translocation, which is essential for TGF-β1-induced HSCs activation.

### TGF-β1-induced SphK1 up-regulation is HuR-dependent

Given that HuR was positively correlated with SphK1 in liver tissues, the relationship between HuR and SphK1 was verified during HSCs activation. We found that TGF-β1-induced SphK1 up-regulation was diminished, while cytoplasmic accumulation of HuR was blocked by LMB treatment (Fig. 3b,d).

**Figure 4:**
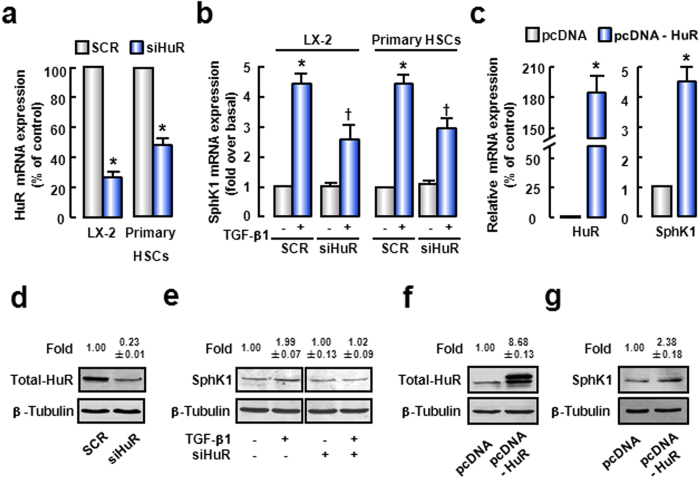
HuR mediates the up-regulation of SphK1 induced by TGF-β1. (**a,b**) LX-2 or primary mouse HSCs were transfected with SCR siRNA or HuR siRNA. 48 hours later, cells were treated with TGF-β1 for another 24 hours. (**a**) The mRNA expression of HuR was detected to confirm the efficiency of HuR knockdown. (**b**) SphK1 mRNA level was also evaluated. (**c**) Cells were transfected with pcDNA or pcDNA-flag-HuR plasmids. 48 hours later, qPCR was performed to assess HuR or SphK1 mRNA level, respectively. (**d,e**) Cells described in Fig. 4a were collected and protein levels of HuR (**d**) and SphK1 (**e**) were evaluated. (**f,g**) Cells described in Fig. 4c were collected and protein level of HuR (**f**) or SphK1 (**g**) was evaluated. The cropped blots are used in the figure and full-length blots are presented in Supplementary Figure 7. The values were the mean intensity normalized of each band (fold over basal). Data are presented as the means ± SEM derived from at least three independent experiments. *P < 0.05, versus untreated control cells. ^†^*P* < 0.05, versus cells treated with TGF-β1 alone.

To further investigate the role of HuR in TGF-β1-induced SphK1 up-regulation, LX-2 were transfected with specific HuR siRNA. As expected, HuR mRNA and protein expressions were decreased by HuR siRNA (Fig. 4a, left pannel and d). Furthermore, up-regulation of SphK1 induced by TGF-β1 was significantly attenuated after HuR silencing (Fig. 4b, left pannel and e). It was suggested that HuR was an important mediator of TGF-β1-induced SphK1 expression. The primary HSCs from normal mouse livers were used to confirm our conclusions. In primary HSCs, HuR knockdown blocked TGF-β1-induced SphK1 mRNA expression (Fig. 4b, right pannel). We also validated this conclusion by HuR overexpression. In cells transfected with pcDNA-HuR plasmids, HuR mRNA and protein levels were increased (Fig. 4c, left pannel and f). At the same time, SphK1 mRNA and protein levels were also significantly up-regulated after HuR over-expression (Fig. 4c, right pannel and g). These data suggest that HuR is involved in TGF-β1-induced up-regulation of SphK1.

**Figure 5:**
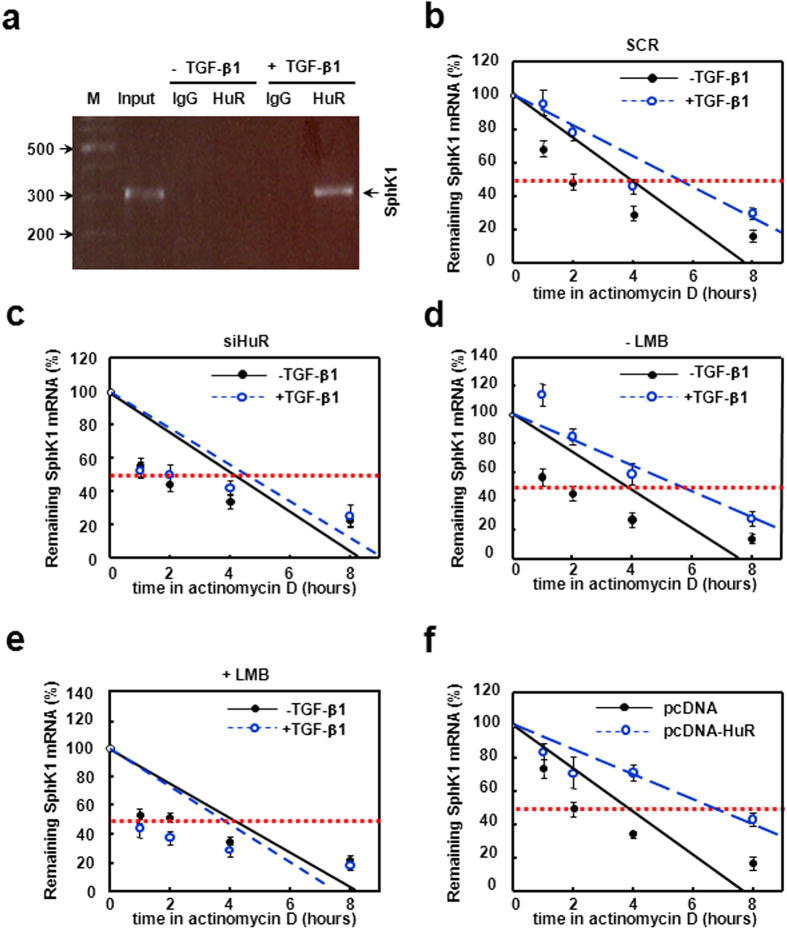
TGF-β1 promotes HuR binding to SphK1 mRNA and prolongs half-life of SphK1 mRNA. (**a**) LX-2 treated with or without TGF-β1 was collected. RIP experiment was performed as described in methods. SphK1 mRNA was measured by qRT-PCR, and the PCR products were size-fractionated in a 2% agarose gel. LX-2 were transfected with SCR siRNA (**b**) or HuR siRNA (**c**) before TGF-β1 treatment for 24 hours, the half-life of SphK1 mRNA was determined by ActD pulse-chase experiments. LX-2 was pre-treated without (**d**) or with (**e**) LMB for 2 hours before TGF-β1 treatment, and SphK1 mRNA half-life was examined. (**f**) Cells were transfected with pcDNA-flag-HuR plasmids and the half-life of SphK1 mRNA was tested. Data are presented as the means ± SEM derived from at least three independent experiments.

Next, we performed RIP to confirm the interaction between HuR protein and SphK1 mRNA *in vitro*. The results showed a significant interaction between HuR and SphK1 mRNA in the presence of TGF-β1, as a significant amount of SphK1 PCR product was amplified in anti-HuR IP complex. RIP using control IgG showed undetectable signals (Fig. 5a). This result suggests that TGF-β1 promotes the binding ability of HuR to SphK1 mRNA.

Since HuR exerts its functions mainly by affecting mRNA stability, we next measured half-life of SphK1 mRNA. As shown in Fig. 5b, TGF-β1 caused a substantial raise in SphK1 mRNA stability (from ~4.0 to ~5.8 hours). Meanwhile, silence of HuR blocked the effect of TGF-β1 on SphK1 mRNA half-life (Fig. 5c). The TGF-β1-induced prolongation of SphK1 mRNA half-life was also lost when HuR cytoplasmic accumulation was inhibited by LMB, (Fig. 5d,e). Conversely, the half-life of SphK1 mRNA in pcDNA-HuR plasmids-transfected cells was significantly longer than that in empty vector-transfected cells (from ~4.0 to ~6.5 hours) (Fig. 5f). These results demonstrate that TGF-β1 up-regulates SphK1 expression by stabilizing the SphK1 mRNA via HuR.

Taken together, these data suggest that HuR binds to SphK1 mRNA and enhances the stability of SphK1 mRNA during liver fibrosis.

### HuR mediates HSC activation through SphK1

**Figure 6:**
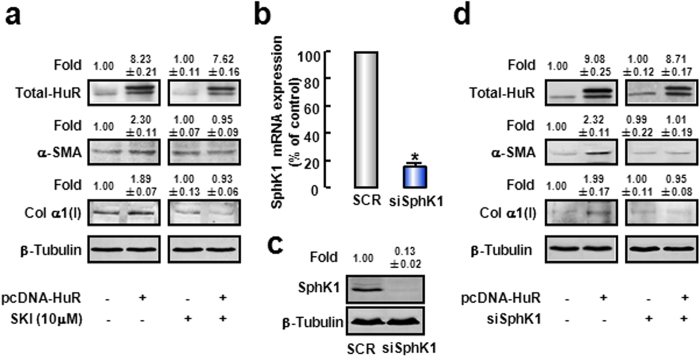
HuR stimulates activation of HSCs via SphK1. (**a**) LX-2 was treated with SKI before transfected with pcDNA-flag-HuR plasmids, α-SMA and Col α1(I) protein expressions were evaluated. The cropped blots are used in the figure and full-length blots are presented in Supplementary Figure 8a. Cells were co-transfected with SphK1 siRNA and pcDNA-flag-HuR plasmids, mRNA (**b**) and protein (**c**) levels of SphK1 were examined to confirm the efficiency of SphK1 knockdown. α-SMA and Col α1(I) protein expressions (**d**) were detected. The cropped blots are used in the figure and full-length blots are presented in Supplementary Figure 7b. β-Tubulin levels were assessed as a loading control. The values were the mean intensity normalized of each band (fold over basal). Data are presented as the means ± SEM derived from at least three independent experiments. *P < 0.05, versus the control cells.

Next, we testified whether HuR mediated activation of HSCs via SphK1. α-SMA and Col α1(I) protein levels were significantly up-regulated when cells were transfected with pcDNA-HuR plasmids (Fig. 6a, left panel). Then SKI, a SphK1 pharmacological inhibitor^28^, was used to treat cells. Western blot Results showed that SKI obviously attenuated the up-regulations of α-SMA and Col α1(I) expressions stimulated by HuR overexpression (Fig. 6a, right panel). We also employed SphK1 siRNA to confirm function of SphK1. After verified the effect of SphK1 siRNA (Fig. 6b,c), we co-transfected cells with both SphK1 siRNA and pcDNA-HuR plasmids. Similar to effects of SKI, HuR-stimulated increases of α-SMA and Col α1(I) expressions were significantly inhibited in SphK1 knockdown cells (Fig. 6d). Altogether, these data prove that HuR stimulates activation of HSCs via SphK1.

### Both HuR and SphK1 are required for TGF-β1-induced HSC activation

**Figure 7:**
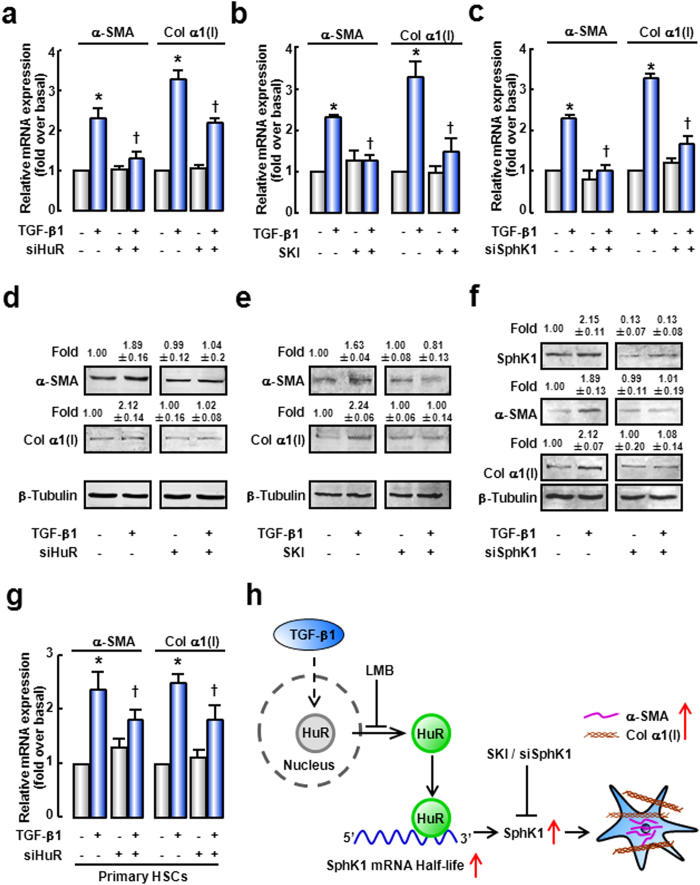
Both HuR and SphK1 are involved in TGF-β1-induced activation of HSCs. LX-2 were transfected with specific siRNA of HuR or SphK1, or pre-incubated with SphK1 inhibitor SKI before TGF-β1 treatment. mRNA levels of α-SMA and Col α1(I) in response to TGF-β1 in the presence of HuR siRNA (**a**), SKI (**b**), or SphK1 siRNA (**c**) were evaluated by qRT-PCR. Protein expressions of α-SMA and Col α1(I) in response to TGF-β1 in the presence of HuR siRNA (**d**), SKI (**e**), or SphK1 siRNA (**f**) were detected by Western blot. The cropped blots are used in the figure and full-length blots are presented in Supplementary Figure 8b–d. β-Tubulin levels were assessed as a loading control. The values were the mean intensity normalized of each band (fold over basal). (**g**) Primary mouse HSCs were transfected with specific siRNA of HuR before TGF-β1 treatment. mRNAs of α-SMA and Col α1(I) were observed. (**h**) Scheme of HSCs activation. Data are presented as the means ± SEM derived from at least three independent experiments. *P < 0.05, versus the control cells without treatment. ^†^*P* < 0.05, versus cells treated with TGF-β1 alone.

To further investigate the role of HuR/SphK1 in TGF-β1-induced HSC activation, SphK1 inhibitor (SKI) and specific siRNA of HuR or SphK1 were employed. mRNA and protein expressions of α-SMA and Col α1(I) were significantly up-regulated as a response to TGF-β1 (Fig. 7a,d). However, silencing of HuR significantly attenuated TGF-β1-induced up-regulations of α-SMA and Col α1(I) expressions (Fig. 7a,d). To rule out the possible off-target effects, we designed another siRNA targeting HuR (position at 3′UTR), and observed similar results (Supplementary Figure 4). Similarly, in primary mouse HSCs, HuR knockdown blocked TGF-β1-induced HSC activation (Fig. 7g).

Moreover, the effects of TGF-β1 on α-SMA and Col α1(I) expressions were prevented when SphK1 activity was inhibited by SKI (Fig. 7b,e). Similar result was observed when SphK1 expression was silenced by siRNA (Fig. 7c,f). These data demonstrate that both HuR and SphK1 are involved in TGF-β1-induced HSC activation.

## Discussion

In the present study, we demonstrate the crucial role of HuR in TGF-β1-induced SphK1 up-regulation and HSCs activation. TGF-β1 triggers HuR cytoplasmic accumulation, and promotes the binding between HuR and SphK1 mRNA. After binding to HuR, SphK1 mRNA stability is increased, and SphK1 expression is up-regulated. HSCs are activated and characterized by up-regulation of α-SMA and Col α1(I) (Fig. 7h). Our study suggests that HuR is an important player in TGF-induced activation of HSCs and might be a useful therapeutic target for liver fibrosis.

In earlier studies, HuR has been proved to participate in the fibrosis of cardiovascular system and kidney^29^,^30^. However, little research focuses on the role of HuR in liver fibrosis. A recent report demonstrates that HuR expression is highly increased in activated HSCs, and silence of HuR in BDL mice results in a decrease of histological liver damage and fibrosis^24^. Consistent with these results, we also confirm that HuR contributes to HSCs activation and liver fibrogenesis. Firstly, we found HuR mRNA was significantly increased in mouse fibrotic livers. Secondly, we observed the positive correlations between HuR and α-SMA or Col α1(I) mRNA expression, respectively. Finally, silence of HuR in LX-2 significantly reduced the up-regulation of α-SMA and Col α1(I) expressions induced by TGF-β1, while HuR overexpression significantly increased α-SMA and Col α1(I) levels.

SphK1 plays a crucial role in human liver damage and fibrosis^2^,^4^. Meanwhile, TGF-β1 up-regulates SphK1 expression and activity in a variety of cells, such as human fibroblasts, podocytes, mouse cardiac fibroblasts, and murine skeletal myoblasts^4^,^11^,^12^,^31^. However, the molecular mechanism under this process is still not fully elaborated. In our previous study, we find that TGF-β1 evokes the activity of SphK1 in a TGF-β1 receptor-dependent manner^8^. Here, our data establish a novel molecular mechanism by which TGF-β1 up-regulates SphK1 expression. It proves that TGF-β1 also regulates SphK1 expression via HuR at the post-transcriptional level. In our research, TGF-β1 obviously prolongs the half-life of SphK1 mRNA and increases SphK1 expression, while silence of HuR attenuates these effects.

HuR is involved in fibrogenesis by affecting target gene expression. For instance, HuR participates in angiotensin II-induced renal fibrosis by stabilizing plasminogen activator inhibitor-1 and cyclooxygenase-2 mRNAs^16^. Silencing HuR leads to suppression of matrix metalloproteinase-9 mRNA expression and activity in NIH3T3 cells^30^. Here, we report SphK1 as a new target gene of HuR involved in liver fibrogenesis.

It should be noticed that the effect of HuR knockdown was not as strong as SphK1 knockdown and the effect of HuR knockdown on SphK1 was smaller than that on α-SMA (Fig. 7). There might be two reasons: (1) The expression of SphK1 is regulated by different mechanisms. In our study, we proved that HuR regulated SphK1 expression in a post-transcriptional regulation. But SphK1 expression also can be regulated at transcriptional level. For example, nerve growth factor increases SphK1 expression by inducing the binding of transcription factor specificity protein 1 to the promoter of SphK1 gene in the neuronal cells^32^. A recent report reveals that Smad signaling pathway involved in the TGF-β1-dependent SphK1 expression^11^. Our previous study also reported that TGF-β1 increases SphK1 activity via phosphorylating SphK1 protein^8^. At the same time, there are many studies which have proved that SphK1 plays a crucial role in TGF-β1-induced effects^8^,^12^. For these reasons, the effects of SphK1 knockdown might be more efficient than HuR knockdown. (2) TGF-β1 is a major profibrogenic cytokine which performs its functions via different manners. In this study, we proved that TGF-β1 could activate HSCs by affecting HuR cytoplasmic translocation. But HuR is not a unique downstream protein which can be activated by TGF-β1. For example, it has been proved that Smad signaling pathway is a classic pathway which is activated by TGF-β1/TGF-β1 receptor and plays an important role in HSCs activation^33^. Meanwhile, HuR also regulates fibrogenic response via other target genes. A research about cardiac fibroblasts reveals that HuR involves in TGF-β1-induced fibrogenic response by regulating the expression of TGF-β1 via a positive feedback circuit^12^. These might be the reason why the effect of HuR knockdown on SphK1 expression is lower than α-SMA expression.

Accumulating evidences have shown that there are several mechanisms by which HuR regulates target gene expression. First, HuR can stabilize a majority of target mRNAs by prolonging the half-life of mRNAs. For example, HuR stabilizes vascular endothelial growth factor mRNA in inflammatory angiogenesis and tumor angiogenesis^17^. In addition, IL-17 mRNA stability is significantly reduced when HuR is knocked out in Th17 cells^34^. Second, HuR affects the translation of target mRNAs. It has been reported that HuR enhances the translation of the ATP-binding cassette transporter A1 in human hepatic and monocytic cells^22^. Another study reveals that HuR inhibits p27 translation after binding to 5′UTR of p27 mRNA^19^. Third, HuR can regulate subcellular distribution of target mRNAs. For instance, HuR affects the nucleo-cytoplasmic translocation of CD83 mRNA through APRIL (a known protein ligand of HuR)^35^. Surprisingly, earlier studies also reveal that HuR destabilizes several target mRNAs, such as p16 and nucleophosmin^36^,^37^. In present study, we found that HuR bound to SphK1 mRNA when TGF-β1 existed, and prolonged SphK1 mRNA half-life in human HSCs. Silencing HuR abrogated the effect of TGF-β1 in prolonging SphK1 mRNA half-life, and overexpression of HuR stabilized SphK1 mRNA. We established that SphK1 expression was up-regulated by HuR in the classic regulatory manner. Our results are consistent with a current study, which reveals that HuR participates in up-regulation of SphK1 expression induced by v-Src in NIH3T3 cells^26^.

The stimulus-dependent cytoplasmic accumulation of HuR is considered as the initial and crucial step of HuR-modulated mRNA stabilization, since HuR is predominantly located in the cell nucleus^15^. In present study, we confirmed that TGF-β1 exerts its functions by driving cytoplasmic accumulation of HuR. When the translocation of HuR was prevented by LMB, TGF-β1-induced up-regulation of α-SMA and Col α1(I) expressions were marked inhibited. Previous studies have proved that there are several signaling pathways involved in the accumulation of HuR in cytoplasm. For instance, TGF-β1 increases cytoplasmic fraction of HuR in a p38MAPK dependent manner in cardiac fibroblasts, since inhibition of p38MAPK by specific inhibitor or silence of p38MAPK almost blocks the cytoplasmic HuR localization^25^. Besides, researchers have proved that AMP-activated kinase, JNK, ERK, protein kinase C (PKC), and PI3K/Akt signalings are also involved in translocation of HuR to cytoplasm^18^,^24^,^38^,^39^,^40^. Moreover, JNK, ERK, PKC and PI3K/Akt have been proved participating in TGF-β1-induced fibrogenesis^41^,^42^,^43^. For these reasons, further experiments are needed to confirm whether these downstream signaling molecules involve in TGF-β1-triggered HuR cytoplasmic accumulation.

Except our study, there are several other studies on the role of HuR in hepatocytes. HuR involves in cell death and apoptosis, while hepatocytes are exposed to hypoxia or treated with TNF-α^44^,^45^. In addition, both HuR/Methyl-HuR and AUF1 regulate liver de-differentiation, development, and human hepatocellular carcinoma (HCC) progression^46^. Furthermore, in HCC-derived cell lines, elevated level of HuR leads to decrease Fas expression and subsequent resistance to Fas-mediated apoptosis^47^. Meanwhile, the cytoskeletal inhibitors latrunculin A and blebbistatin exert antitumorigenic properties by interfering with intracellular HuR trafficking^48^. However, it remains undefined whether HuR plays a general role in modulating the TGF-β1 signaling in terms of target genes remains undefined.

In conclusion, we demonstrate that RNA binding protein HuR plays a pivotal role in TGF-β1-stimulated SphK1 expression and HSCs activation. After treatment with TGF-β1, HuR translocates into cytoplasm, binds to SphK1 mRNA, enhances the stability of SphK1 mRNA and finally results in up-regulation of SphK1 expression. This event represents a novel molecular mechanism by which TGF-β1 contributes to liver fibrogenesis.

## Methods

### Materials

TGF-β1 was purchased from PeproTech (London, UK). The Sphingosine Kinase Inhibitor SKI [2-(p-hydroxyanilino)-4-(p-chlorophenyl) thiazole] was acquired from Calbiochem (Bad Soden, Germany). Leptomycin B (LMB) was obtained from Sigma-Aldrich (St Louis, MO). Actinomycin D (ActD) was from Solarbio (Beijing, China). PCR reagents were from Applied Biosystems (Life Technologies, Foster City, CA).

### Mouse Models of Chronic Liver Fibrosis

Mouse models of liver fibrosis were induced by BDL operation or injection of CCl_4_ as previously described^49^. BDL was performed on adult male ICR mice. Sham-operated mice were underwent a laparotomy with exposure, but no ligation of the common bile duct. Mice were sacrificed at 2 weeks of BDL (n = 7 per group). For CCl_4_ treatment, adult ICR mice were treated with 1 μL/g body weight of CCl_4_ diluted (1:9 v/v) in olive oil (OO) by intraperitoneal injections twice per week. The mice were sacrificed at 4 weeks of CCl_4_ treatment (n = 7 per group), always on the day after the last injection. Control animals received vehicle alone. Liver tissues were collected for further experiments. All animal work was approved by the Ethics Committee of Capital Medical University and in accordance with the approved guidelines (approval number: AEEI-2014–131).

### Human liver specimens

Human liver specimens were prepared from Snap-frozen surgical liver resections from 21 patients (13 men, 8 women; mean age, 56 yr; range, 42–69 yr). Normal liver samples were collected from 5 patients undergoing hepatic resection for colorectal metastasis (n = 5). Fibrotic samples (fibrosis stage: F2–4) were obtained from 16 livers of patients undergoing liver transplantation. Fibrosis was consecutive to chronic hepatitis C virus (n = 4) or hepatitis B virus (n = 10) infections, and alcohol induced liver disease (n = 2). All tissues were obtained with donor consent and the approval of the Capital Medical University Ethics Committee (approval number: 2011SY08).

### Cell Culture

LX-2 were cultured in Dulbecco’s modified Eagle’s medium (Gibco, Life Technologies, Foster City, CA) supplemented with 10% fetal bovine serum, 100 mg/mL streptomycin, 100 units/mL penicillin and L-Glutamine (2 mmol/L), at 37 °C in 5% CO_2_. The preparation of primary HSCs from liver is performed as described^50^. Unless otherwise indicated, cells prepared for experiments were cultured in serum-free medium for 12 hours, followed by TGF-β1 (10 ng/mL) stimulation, 24 hours later, cells were collected. SKI (10 μmol/L) was added 1 hour before TGF-β1 treatment, while LMB (10 nmol/L) was exerted 2 hours before.

### RNA Interference and Transient Transfection

Small interfering RNA (siRNA) sequences specifically targeting human HuR (position at 649–669, 5′-AAGAGGCAAUUACCAGUUUCA-3′; 3′UTR, 5′-AACGACTCAATTGTCCCGATA-3′), mouse HuR (position at 1430–1450, 5′-CCCACAAATGTTAGACCAATT-3′), human SphK1 (4390824-s16957, Ambion, Austin, TX) or scramble siRNA (SCR siRNA, 4390843, Ambion, Austin, TX) was synthesized respectively, and was transfected using Lipofectamine RNAiMAX (Invitrogen, Grand Island, NY) as recommended by the manufacturer’s instructions. pcDNA-flag-HuR plasmid was a kind gift from Prof. Wengong Wang of Peking University. LX-2 were seeded overnight and transfected with the pcDNA-flag-HuR plasmid or empty plasmid, using Lipofectamine 2000 (Invitrogen) in accordance with the manufacturer’s instructions. 48 hours later, cells were collected for further analysis.

### qRT-PCR and mRNA Half-life Measurement

Total RNA of LX-2 and liver tissues were extracted and qRT-PCR was performed as a study described^49^. Sequences of primers used in qRT-PCR as follow: 18S rRNA sense: GTAACCCGTTGAACCCCATT, anti-sense: CCATCCAATCGGTAGTAGCG, GAPDH sense: CGAGTCAACGGATTTGGTGGTAT, anti-sense: AGCCTTCTCCATGGTGAAGAC, TGF-β1 (mouse) sense: TGCGCTTGCAGAGATTAAAA, anti-sense: TCACTGGAGTTGTACGGCAG, HuR (human) sense: ACCAGGCGCAGAGATTCA, anti-sense: GGTTGTAGATGAAAATGCACCAG, HuR (mouse) sense: ATGCTGCTGAACAGACTTCG, anti-sense: TGTCTAATGGTTATGAAGACCACA, Col α1(I) (human) sense: AGGTCCCCCTGGAAAGAA, anti-sense: AATCCTCGAGCACCCTGA, Col α1(I) (mouse) sense: AGGGCGAGTGCTGTGCTTT, anti-sense: CCCTCGACTCCTACATCTTCTGA, α-SMA (human) sense: CCCTGAAGTACCCGATAGAACA, anti-sense: GGCAACACGAAGCTCATTG, α-SMA (mouse) sense: ATGCTCCCAGGGCTGTTTT, anti-sense: TTCCAACCATTACTCCCTGATGT, SphK1 (human) sense: TTGAACCATTATGCTGGCTATG, anti-sense: GGTGTCTTGGAACCCACTCTT, SphK1 (mouse) sense: TGACTGTCCATACCTGGTTCATG, anti-sense: CATCAGCTCTCCATCCACAGAA. Probes (Applied Biosystems) were as follows: human SphK1: hs00184211_m1; mouse SphK1:Mm00448841_g1. To measure the half-life of SphK1 mRNA, cells were incubated in culture medium added Actinomycin D (ActD, the RNA polymerase II inhibitor, 2 μg/mL). Total RNA was isolated at the indicated time.

### Western Blot Analysis

Whole-cell or cytoplasmic extracts were prepared as a previous study described^51^. Western blot analysis was carried out with standard procedures and followed primary antibodies against HuR (1:1000; Santa Cruz Biotechnology, Santa Cruz, CA), SphK1 (1:1000; Cell Signaling, Beverly, MA), α-SMA (1:1000; Sigma-Aldrich, St Louis, MO) or Col α1(I) (1:250; Santa Cruz Biotechnology). IRDyeTM 800-conjugated Goat anti-rabbit IgG or goat anti-mouse IgG (1:10,000, LI-COR Biosciences, Lincoln, NE) was applied appropriately as secondary antibodies. Protein expression was visualized and quantified by the LI-COR Odyssey® Imaging System and Odyssey® software (LI-COR Biosciences, Lincoln, NE), respectively. Results were normalized relative to β-Tubulin (1:1000; Cell Signaling, Beverly, MA) expression to correct for variations in protein loading and transfer.

### Immunofluorescence and High Content Analysis

HuR immunofluorescence and High Content Analysis were performed as described previously^10^,^52^. Immunofluorescent detection of HuR in liver tissue was performed using a Vector M.O.M. (mouse-on-mouse) immunodetection kit (Vector Laboratories) and a monoclonal antibody to HuR (1:100, Santa Cruz Biotechnology, CA). High Content Analysis is an application of automated microscopy and image analysis to cell biology. The cells plated in 96-well plates (Corning, NY) were imaged on Thermo Scientific Cell Insight personal cell imaging (PCI) platform (Cellomics, Inc., Thermo Fisher Scientific Inc., Waltham, MA), with a × 20 objective using the Thermo Scientific Cellomics iDEV Software. At least 36 images were acquired by the software and total fluorescence intensity of each well was analyzed. For negative controls, sections or cells were processed the same way, except omitting incubation with the primary antibody.

### Isolation of Non-parenchymal Cells of Mouse Liver

Non-parenchymal cells of mouse liver were isolated as our previous study described^53^. Briefly, livers were perfused with 20 mL of PBS, minced with scissors and digested for 30 minutes with collagenase type IV at 37 °C with gentle shaking. Digested extracts were pressed through 70-mm cell strainers to achieve single cell suspensions. The cell suspension was subjected to density gradient (Histopaque-1077) centrifugation at 2000 rpm for 20 minutes. The cells were collected from the interface after centrifugation, washed twice with PBS.

### RIP

To assess the association of endogenous HuR with target mRNAs, RIP assay was performed as a previous study described^54^. In brief, cells with or without treatment were collected, lysed in polysome lysis buffer supplemented with protease inhibitor and RNase inhibitor. The supernatants were incubated with protein G-Sepharose beads pre-coated with anti-HuR or control IgG for 2 hours at room temperature. After removing DNA and proteins, total RNA was extracted from the supernatants and examined by qRT-PCR. PCR products were size-fractionated in a 2% agarose gel to detect the presence of SphK1.

### Statistical Analysis

Data are presented as means ± standard error of the mean (SEM). Differences between groups were evaluated using a two-sided Student’s t-test. Correlations between HuR and fibrosis markers expressions in liver tissue were assessed with the Pearson correlation coefficient. P < 0.05 was considered significant. All results were confirmed in at least three independent experiments.

## Additional Information

**How to cite this article**: Ge, J. *et al.* Essential Roles of RNA-binding Protein HuR in Activation of Hepatic Stellate Cells Induced by Transforming Growth Factor-β1. *Sci. Rep.* **6**, 22141; doi: 10.1038/srep22141 (2016).

## Supplementary Material

Supplementary Information

## Figures and Tables

**Table 1:** The ratio of cytoplasmic to nuclear HuR protein expression (Cyto/Nuc) in cells treated with/without LMB in the presence or absence of TGF-β1.

Sample	Fold		Cyto/Nuc
	Cyto	Nuc	
Control	1.00 ± 0.00	1.00 ± 0.00	1.00 ± 0.00
TGF-β1	1.96 ± 0.13*	0.47 ± 0.05*	4.18 ± 0.14*
LMB	1.01 ± 0.11	1.02 ± 0.12	0.99 ± 0.08
LMB/TGF-β1	0.99 ± 0.09^†^	1.03 ± 0.05^†^	0.96 ± 0.12^†^

Numerical value: folds over controls. Data are presented as the means ± SEM derived from at least three independent experiments. *P < 0.05 compared with controls. ^†^*P* < 0.05, compared with TGF-β1 treatment alone.
