# Supplementary material for: Essential Roles of RNA-binding Protein HuR in Activation of Hepatic Stellate Cells Induced by Transforming Growth Factor-β1
Source: Sci Rep. 2016 Feb 25;6:22141. doi: 10.1038/srep22141 (PMC4766441; doi:10.1038/srep22141)
Supplement: Supplementary Information [file srep22141-s1.pdf]

Essential Roles of RNA-binding Protein HuR in Activation of Hepatic Stellate Cells Induced by Transforming Growth Factor-β1

Jingjing Ge[1], Na Chang[1], Zhongxin Zhao, Lei Tian, Xianghui Duan, Lin Yang, Liying Li*

Supplementary Information

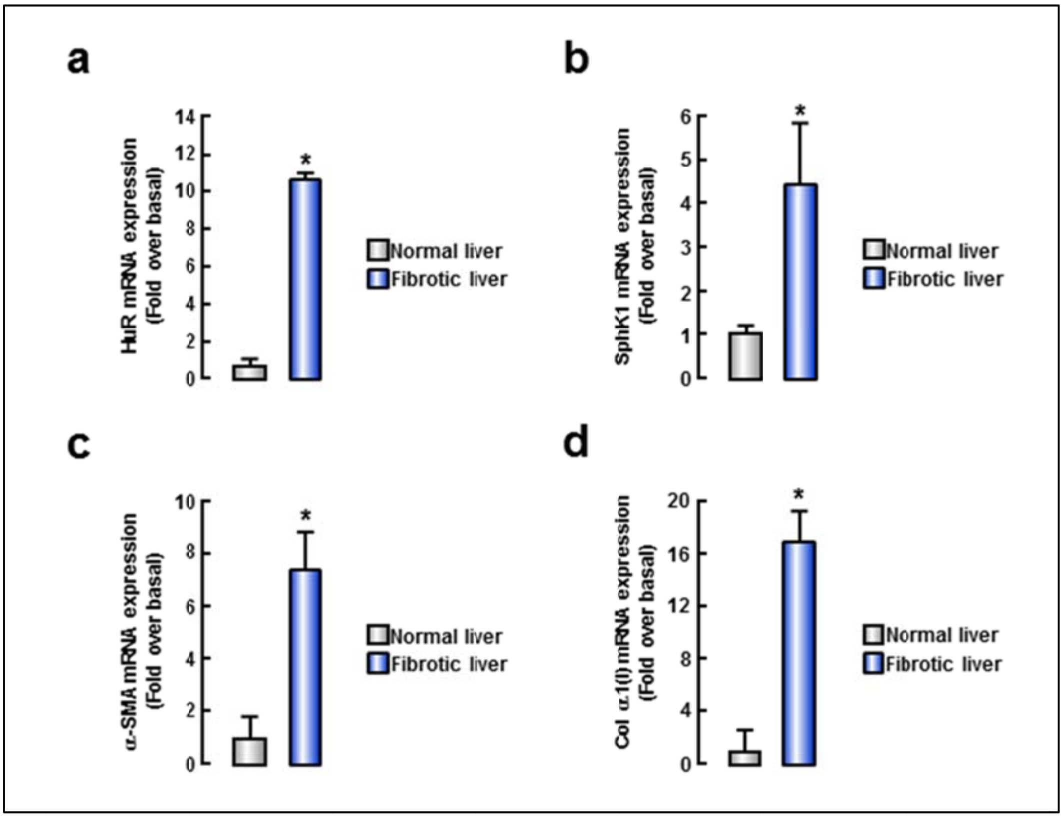

Supplementary Figure 1: HuR is up-regulated in human fibrotic livers. The mRNA expression of HuR (a), SphK1 (b), α-SMA (c), and col α1(I) (d) in human fibrotic livers. Data are presented as the means ± SEM. *$P < 0.05$, versus the normal livers.

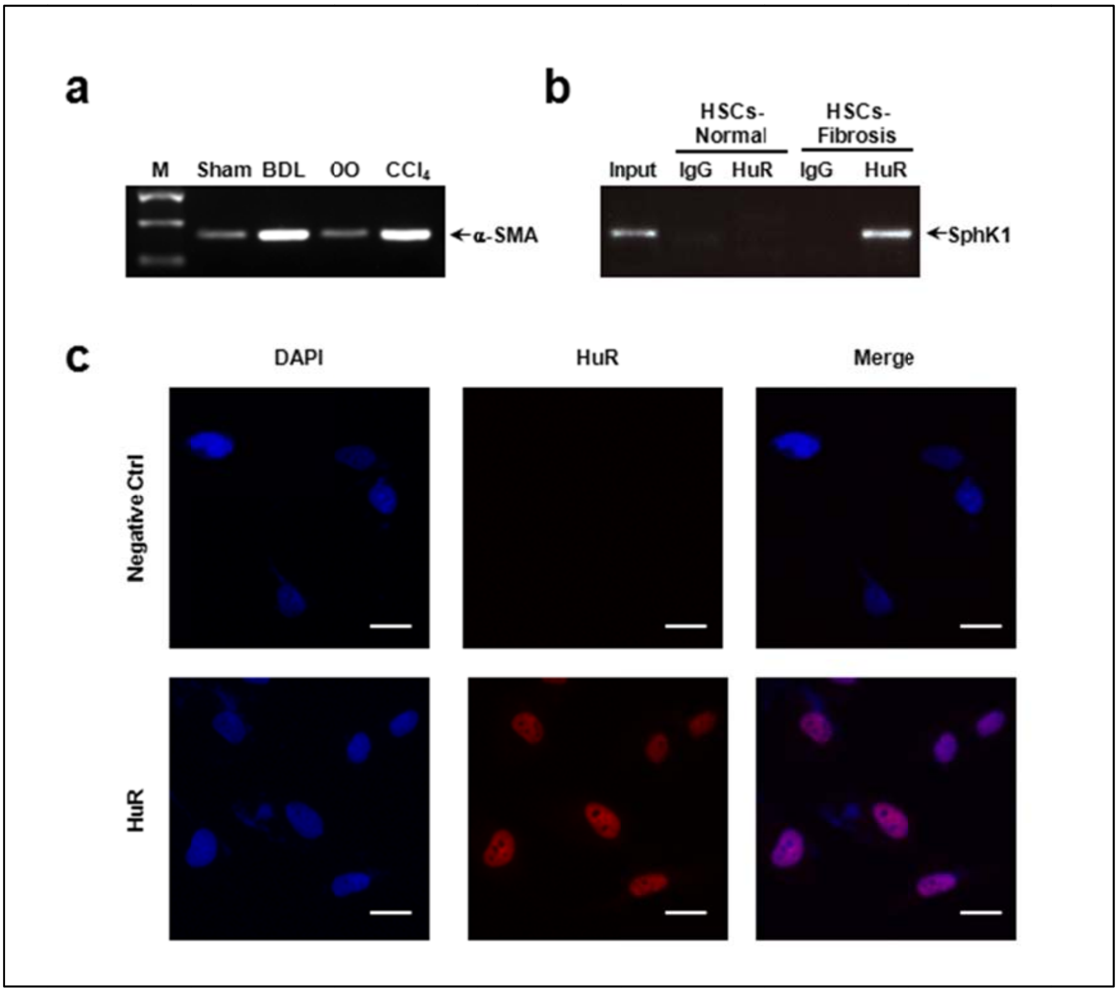

Supplementary Figure 2: HuR binds to SphK1 mRNA in primary mouse HSCs.

(a) The expression of α-SMA was detected in hepatic non-parenchymal cells. (b) Primary mouse HSCs from normal or fibrotic livers were isolated and RIP analysis was performed. SphK1 mRNA was measured by qPCR, and the PCR products were size-fractionated in a 2% agarose gel. The gels are run under the same experimental conditions. (c) The representative images of immunostaining for HuR (red) in primary HSCs, as visualized by immunocytochemical analysis. Cells were co-stained with DAPI to identify nuclei (blue). Scale bars = 25 μm.

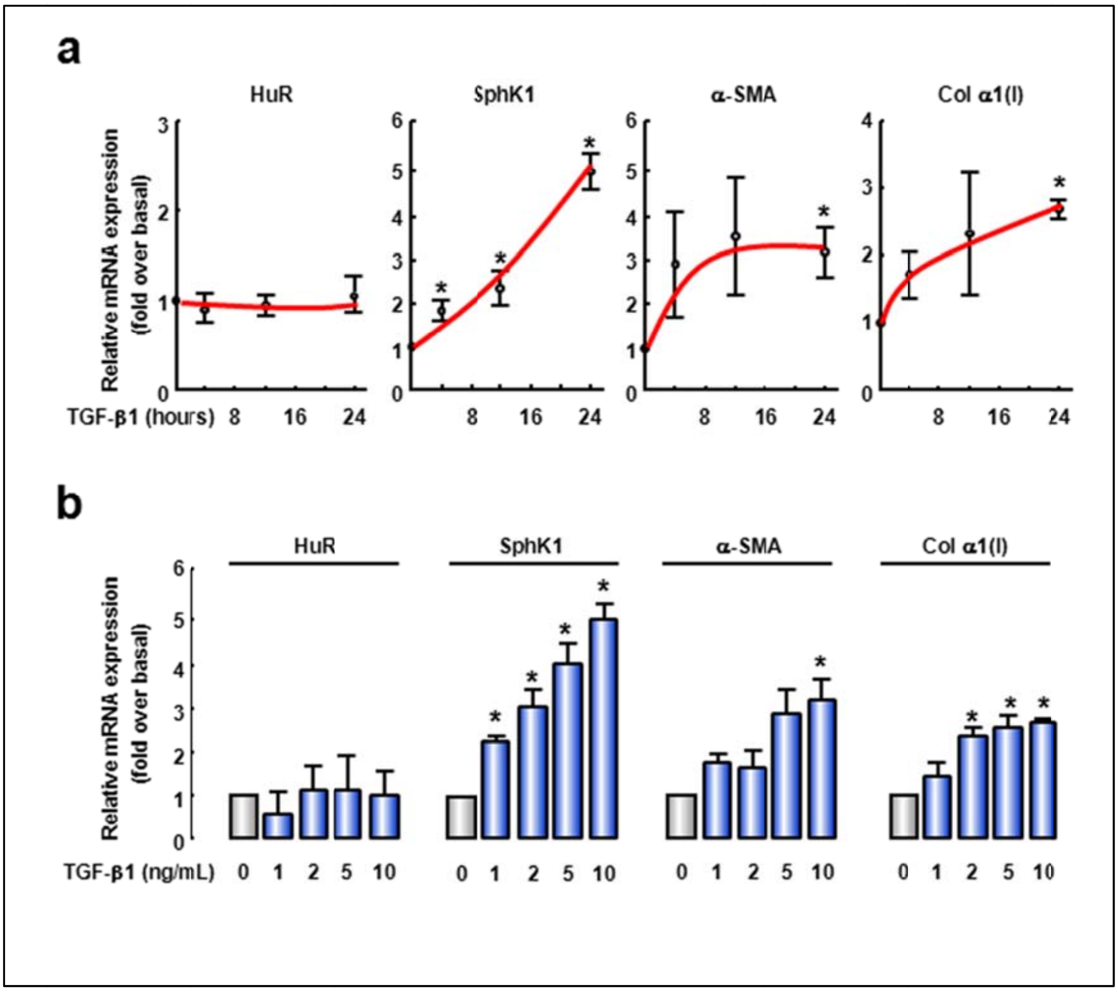

Supplementary Figure 3: TGF-β1 induces SphK1 up-regulation and HSCs activation. LX-2 were treated with 10 ng/mL TGF-β1 and collected at 0, 4, 12, and 24 hours (a) or treated with 0, 1, 2, 5, or 10 ng/mL TGF-β1 for 24 hours (b). mRNA levels of HuR, SphK1, α-SMA and Col α1(I) were detected by qPCR. Data are presented as the means ± SEM derived from at least three independent experiments. **P* < 0.05, versus the control cells without treatment.

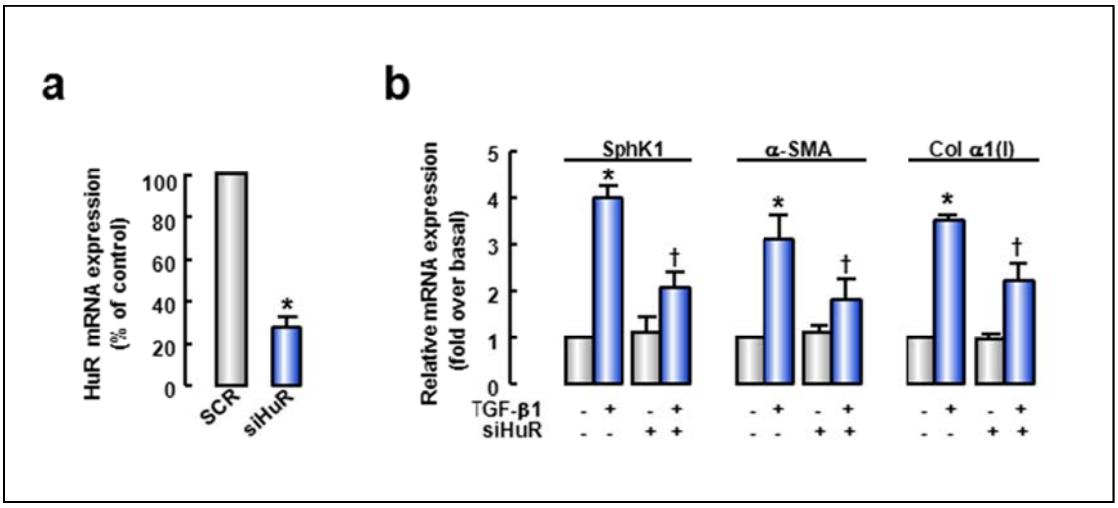

Supplementary Figure 4: Another HuR siRNA verifies the role of HuR in activation of HSCs induced by TGF-β1. LX-2 were transfected with another specific siRNA of HuR (positions 3'UTR), 48 hours later, cells were treated with TGF-β1 for another 24 hours. (a) The mRNA expression of HuR was detected to confirm the efficiency of HuR knockdown. (b) mRNA levels of SphK1, α-SMA and Col α1(I) in response to TGF-β1 in the presence of the new HuR siRNA. Data are presented as the means ± SEM derived from at least three independent experiments. *$P < 0.05$, versus the control cells without treatment. †$P < 0.05$, versus cells treated with TGF-β1 alone.

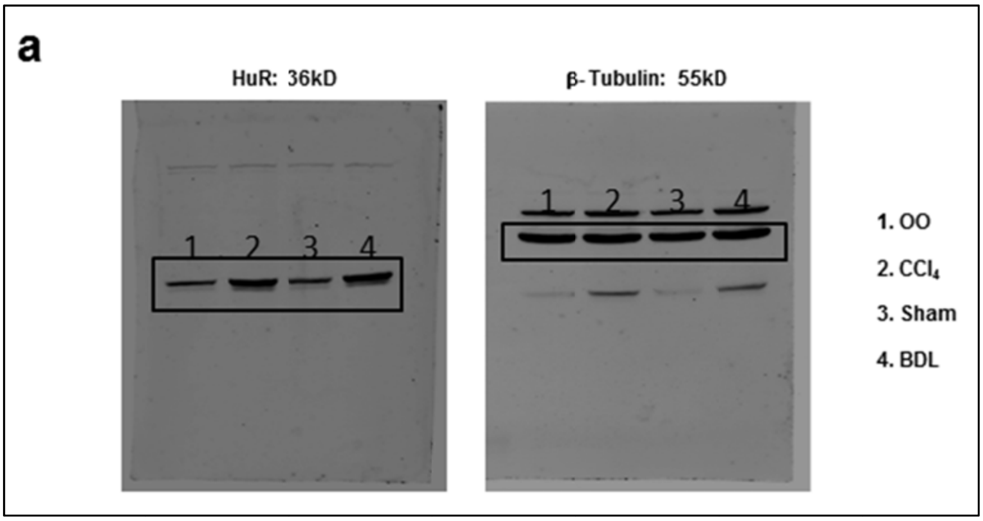

Supplementary Figure 5: The full-length blots of Figure 1. The full-length blots of Figure 1.

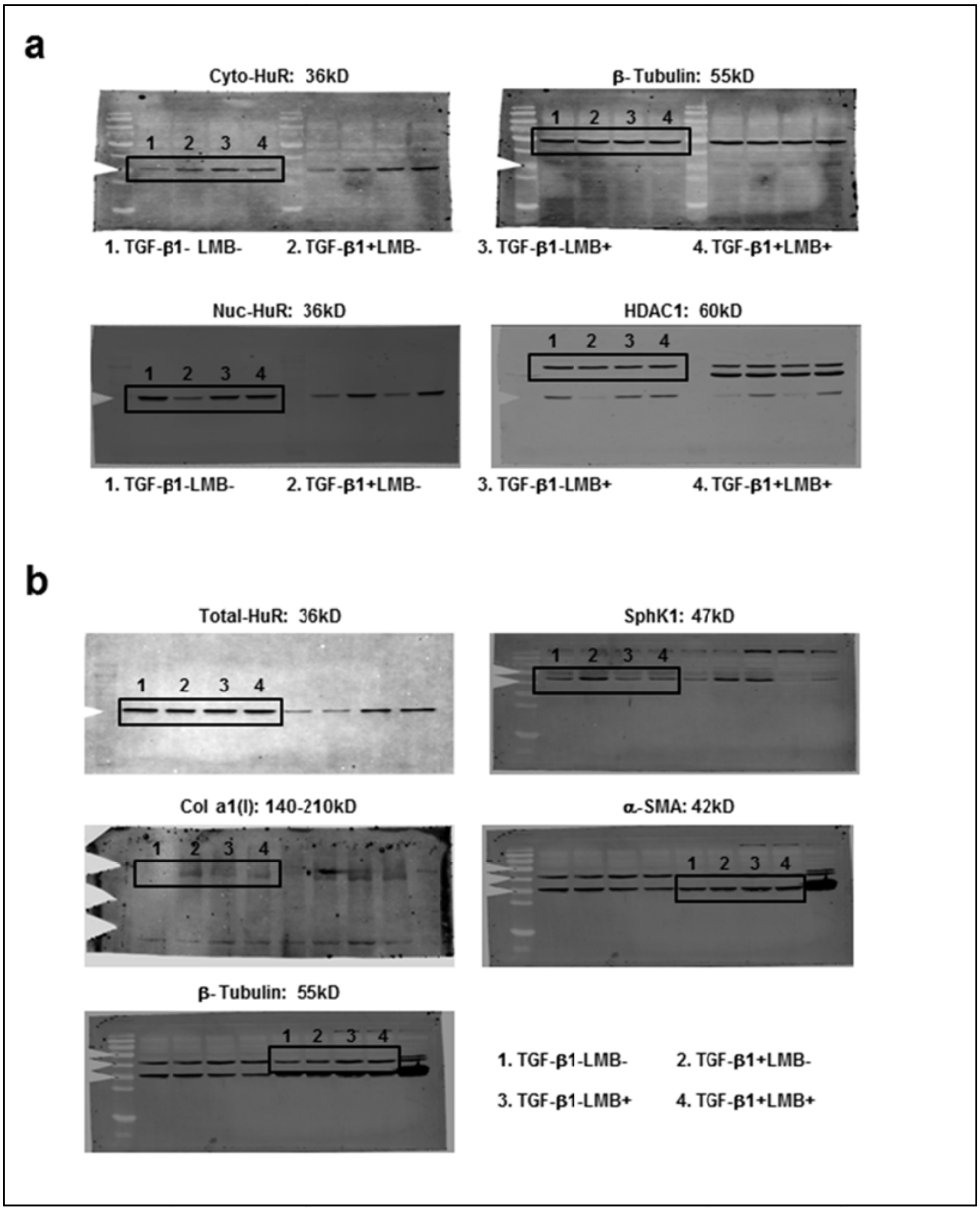

Supplementary Figure 6: The full-length blots of Figure 3. (a) The full-length blots of Figure 3a. (b) The full-length blots of Figure 3b.

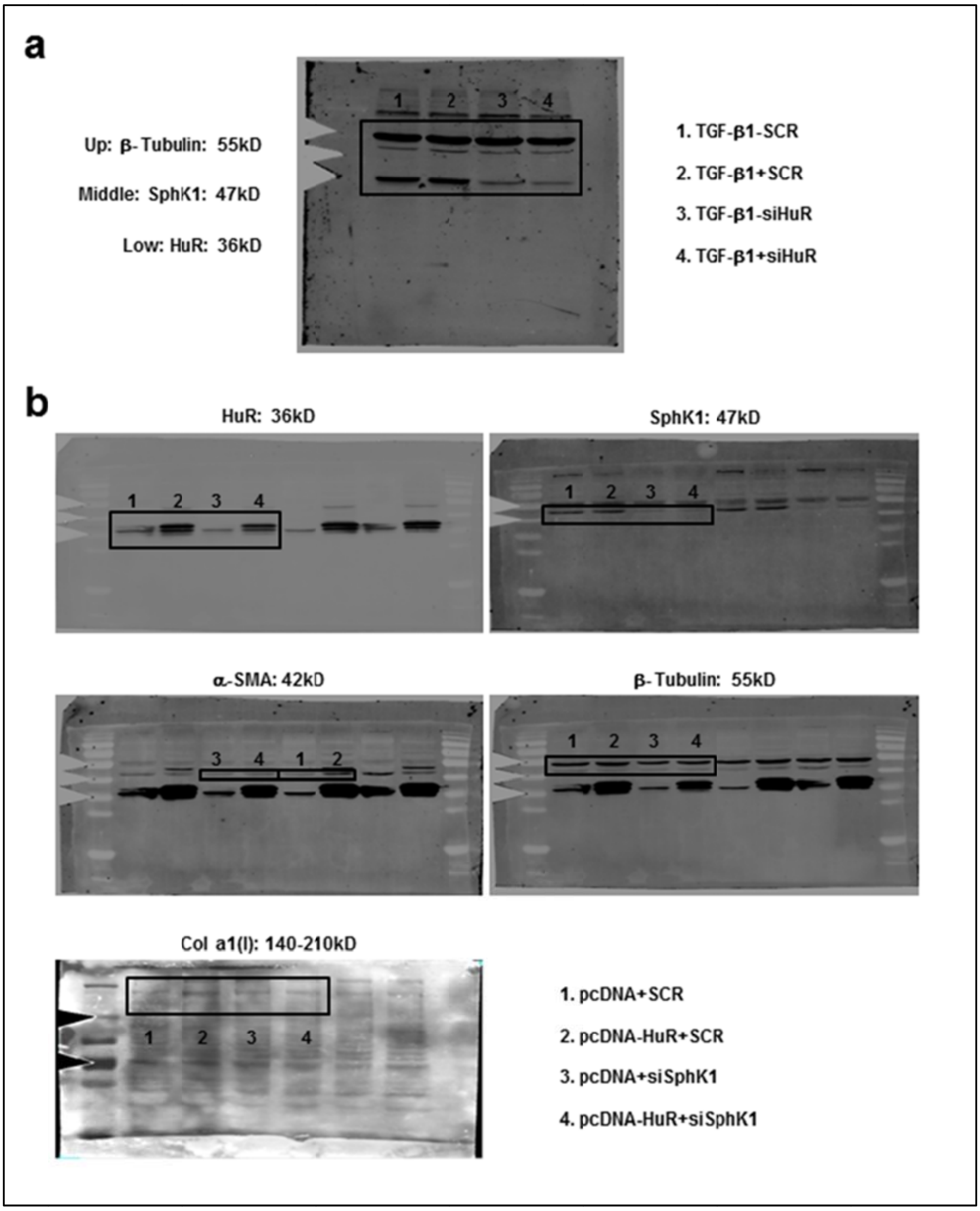

Supplementary Figure 7: The full-length blots of Figure 4, 6c and d. (a) The full-length blots of Figure 4d and e. (b) The full-length blots of Figure 4, 6c and d. Briefly, Band 1-2 are used in Figure 4f and g, band 2-3 are used in Figure 6c and band 1-4 are used in Figure 6d.

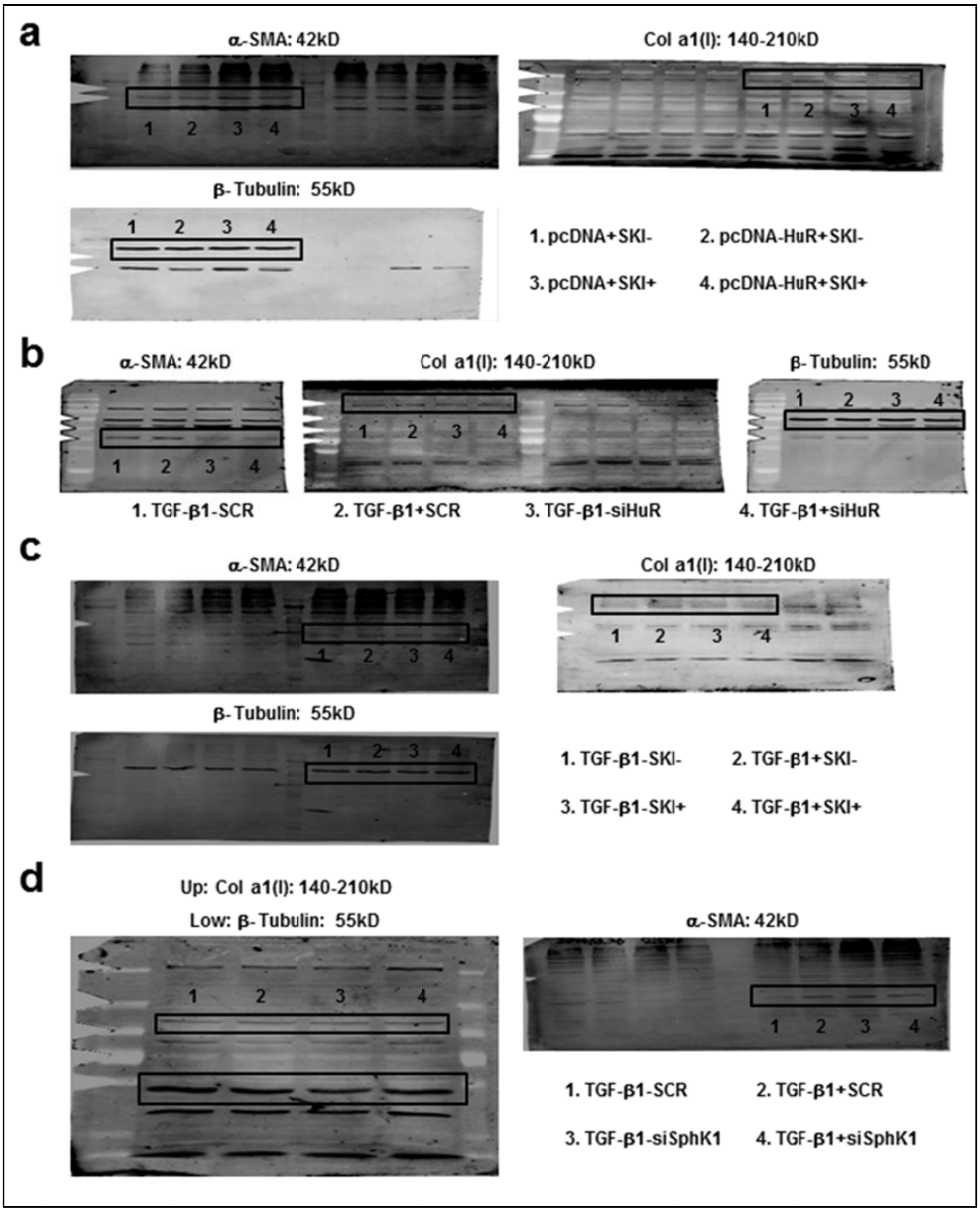

Supplementary Figure 8: The full-length blots of 6a and 7. (a) The full-length blots of Figure 6a. (b) The full-length blots of Figure 7d. (c) The full-length blots of Figure 7e. (d) The full-length blots of Figure 7f.
